# Screening for Severe Illness at Diagnosis Has the Potential to Prevent Early TB Deaths: Programmatic Experience From Karnataka, India

**DOI:** 10.9745/GHSP-D-21-00736

**Published:** 2022-08-30

**Authors:** Hemant Deepak Shewade, Sharath Burugina Nagaraja, Basavarajachar Vanitha, Hosadurga Jagadish Deepak Murthy, Madhavi Bhargava, Anil Singarajipura, Suresh G. Shastri, Bharatkumar Hargovandas Patel, Kajal Davara, Ramesh Chandra Reddy, Ajay M.V. Kumar, Anurag Bhargava

**Affiliations:** a ICMR–National Institute of Epidemiology, Chennai, India.; b Employees’ State Insurance Corporation Medical College and PGIMSR, Bengaluru, India.; c Sri Atal Bihari Vajpayee Medical College and Research Institute, Bengaluru, India.; d Bangalore Medical College and Research Institute, Bengaluru, India.; e Centre for Nutrition Studies, Yenepoya (Deemed to be University), Mangaluru, India.; f Yenepoya Medical College, Yenepoya (Deemed to be University), Mangaluru, India.; g Department of Health and Family Welfare, Government of Karnataka, Bengaluru, India.; h Community Medicine Department, GMERS Medical College, Vadodara, India.; i International Union Against Tuberculosis and Lung Disease (The Union), Paris, France.; j The Union South-East Asia Office, New Delhi, India.

## Abstract

Due to the coronavirus disease (COVID-19) pandemic and its associated response, TB deaths increased for the first time in a decade. In any potentially fatal illness, an assessment of severity is essential. This is not systematically done for adults with TB, mostly due to a lack of policy and/or limited availability of diagnostic and clinical capacity. We developed a screening tool using simple and easily measurable indicators that can be used by paramedical TB program staff to quickly identify people with severe illness. During October–November 2020 in Karnataka, India, the paramedical program staff from 16 districts screened people with TB (aged ≥15 years) notified by public facilities for “high risk of severe illness,” which was defined as the presence of any of the following indicators: (1) body mass index (BMI) ≤14.0 kg/m^2^; (2) BMI ≤16.0 kg/m^2^ with bilateral leg swelling; (3) respiratory rate >24/minute; (4) oxygen saturation <94%; (5) inability to stand without support. In this cohort study, we determined the incidence of program-recorded early deaths (within 2 months) and its association with high risk of severe illness. Of 3,010 people with TB, 1,529 (50.8%) were screened at diagnosis/notification, of whom 537 (35.1%) had a high risk of severe illness. There were 195 (6.5%, 95% CI=5.7, 7.4) early deaths: 59 (30.2%) within a week and 100 (51.3%) within 2 weeks of treatment initiation. The incidence of early deaths was significantly higher among those with high risk of severe illness (8.9%) at diagnosis compared to those without (3.8%) [adjusted relative risk: 2.36 (95% confidence interval=1.57, 3.55)]. To conclude, early deaths were especially high during the first 2 weeks and strongly associated with a high risk of severe illness at diagnosis/notification. Screening for severe illness should be explored as a potential strategy to end TB deaths.

## Abstract (precis)

Despite TB being a potentially fatal disease, severity is not systematically assessed at the start of drug-susceptible TB treatment. We document our experience screening people for severe illness at diagnosis/notification in program settings and the potential impact on reducing early TB deaths.

## Teaser

**Key Findings:**

- Severity is not systematically assessed at the start of TB treatment among adults due to a lack of policy and/or diagnostic and clinical capacity.
- Paramedical TB program staff screened adults with TB for “high risk of severe illness” at notification using a screening tool involving simple and easily measurable indicators.
- Early deaths were seen in 7% of patients; deaths were significantly higher among people with a high risk of severe illness.

**Key Implications:**

- Screening for severe illness should be explored as a potential strategy to end TB deaths.
- Considering the increase in estimated TB deaths due to the coronavirus disease (COVID-19) pandemic, program managers should use this window of opportunity to implement screening for severe illness.

## INTRODUCTION

In 2020, there were an estimated 1.3 million global TB-related deaths, with an estimated case fatality ratio of 13:100.^1^ Due to the coronavirus disease (COVID-19) pandemic response, TB deaths increased for the first time in a decade.^1^ The World Health Organization’s (WHO) End TB targets include a 90% reduction of TB deaths by 2030 and a 95% reduction by 2035 (relative to 2015).^2^^,^^3^

Deaths during the initial months of treatment contribute to most TB deaths and are primarily due to diagnostic delays, the progression of TB to severe disease, and delayed care seeking.^4^^–^^10^ These can be averted by detecting the missing 3.8 million people with TB (including drug-resistant forms and their co-morbidities) as early as possible and treating them appropriately.^1^ Those at higher risk of death (people with TB who are severely ill) should be identified early and provided appropriate inpatient clinical care for severe disease or any serious comorbidities.^11^ In any potentially fatal illness, an assessment of severity is essential. However, this is not implemented systematically among people with TB.

TB deaths during the initial months of treatment contribute to the greatest number of deaths and are primarily due to diagnostic delays, the progression of TB to severe disease, and delayed care seeking.

Globally, there is limited literature on the burden of severe illness at notification and the feasibility of collecting these data in routine program settings. In India, the 2021 National TB Program (NTP) guidance (released after the initiation of this study) categorically recommends severity assessment as soon as possible after diagnosis and referral for inpatient care, if severely ill.^12^. Similar to the tools used by higher-income countries,^13^^,^^14^ this guidance requires clinical, laboratory, and radiological evaluations, which can be challenging in peripheral health institutions (PHIs).^11^

The severity of illness can be screened by evaluation of vital signs, degree of wasting (reflected by BMI), or inability to stand without support (Box).^11^^,^^15^ These easily assessed and interpreted indicators are known risk factors for death.^14^^–^^22^ People who present with any of these indicators may be referred to higher facilities for comprehensive clinical evaluation and inpatient care. The potential impact of this strategy in preventing early deaths (within 2 months) in routine program settings has not been explored.

BOX **Tool to Screen for High Risk of Severe Illness at Notification Among People With TB ^11^^,^^15^^,a^**

If at least 1 of the following is present, then the person with TB is at high risk of severe illness (requires referral for clinical evaluation and assessment for inpatient care):

1. Body mass index (BMI) less than or equal to (≤) 14.0 kg/m^2b^
2. BMI less than or equal to (≤) 16.0 kg/m^2^ with leg swelling^b^
3. Respiratory rate more than (>) 24 per minute^c^
4. Oxygen saturation less than (<) 94%^c^
5. Not able to stand without support (standing with support, squatting, sitting, or bedridden)

^a^ Reprinted from Shewade et al.^23^ under a CC license, with permission from MDPI, © MDPI 2021.

^b^ Very severe undernutrition indicators.

^c^ Respiratory insufficiency indicators (tool adapted from Bhargava et al.^11^

The severity of TB illness can be screened by evaluation of vital signs, degree of wasting (reflected by BMI), or performance status, which are known risk factors for death.

During October–November 2020, in 16 districts in Karnataka (a state in South India), paramedical TB programs and general health staff at PHIs screened people with TB for “high risk of severe illness” at diagnosis/notification (Box).^11^^,^^15^ The findings of the screening process have already been published.^23^ Due to the prevailing COVID-19 pandemic, the coverage of screening was low (51%); however, the time interval from notification to screening was short (only a few days) and data was collected for all indicators in most instances.^23^ In this article, we present the findings related to the incidence of early TB deaths (within 2 months of treatment initiation) and its association with a high risk of severe illness at baseline.

## METHODS

### Study Design and Participants

This was a cohort study involving primary and secondary data. We included all people with TB (age ≥15 years, all forms without known drug-resistant disease at diagnosis) notified by public PHIs in select districts (n=16) in Karnataka (India) between October 15 and November 30, 2020.^23^ We included study participants irrespective of their treatment initiation status or transfer-out status. We excluded adults transferred in from non-study districts.

### Setting

India has the world’s highest TB burden, with an estimated case fatality ratio of 19:100 in 2020 (increased from 17:100 in 2019).^1^^,^^24^ The COVID-19 response-related lockdown possibly contributed to a 21% increase in estimated TB deaths during 2020.^25^ Additional interventions are needed if we are to attain the 2030 WHO End TB targets by 2025.^2^^,^^26^

Karnataka is a state in South India with a population of approximately 64 million and a TB case notification rate of 136 per 100,000 population (as of 2019).^27^ For the 2018 cohort, the treatment success rate was 81% and case fatality was 7%, among the highest reported by a state in India.^27^ The state TB program infrastructure includes 31 districts with subdistrict level administrative units and PHIs (public and private) with at least 1 medical doctor and a designated microscopy center (DMC) for sputum microscopy with a laboratory technician. Due to the COVID-19 pandemic, most public PHIs had a portable pulse oximeter. Each PHI maintains a paper-based register of people with TB, their management, and their treatment outcomes. Each subdistrict level unit has a senior TB treatment supervisor (STS) who updates these details in the NIKSHAY application (a case-based, web-based electronic TB information management system) via a mobile tablet. Each NTP district has a dedicated data entry operator (DEO), and public PHIs in urban areas may have additional staff, called TB health visitors, to support the STS. People with TB receive daily treatment under the direct observation of a health care provider, community volunteer, or family member.

### Data Collection, Variables, and Sources of Data

Due to COVID-19 travel restrictions, the investigators conducted online training for DEOs and STS, who in turn trained the laboratory technicians and TB health visitors during the district’s routine monthly meeting (standard screening procedures are included in Supplement 1).

The laboratory technician or the TB health visitor collected the screening data (primary data) at the first opportunity in a paper-based form (Supplement 2) with the support of a staff nurse or medical doctor. The STS transcribed the details into the Epicollect5 mobile application (an open-access application that allows offline mobile or tablet-based data capture and synchronizes data in the cloud). Before the study period, we piloted this data collection process for 10 days to address unforeseen issues. On December 15, 2020, we extracted the screening data from Epicollect5 and baseline characteristics from NIKSHAY (Supplement 3).

We tracked the program-reported treatment outcome (in treatment, death, loss to follow-up, and transfer to drug-resistant TB care) for each person for up to 2 months. The date of outcome was also extracted. 

### Data Analysis and Statistics

We analyzed the data using EpiData Analysis (v 2.2.2.183 EpiData Association) and STATA version 12.1 (StataCorp) software.

We summarized the distribution of early deaths since beginning treatment (since diagnosis if treatment was not initiated), overall and stratified by exposure status, using incidence proportion, incidence rate (per 100 person-months), and Kaplan-Meier failure curves.

We compared the baseline characteristics with a high risk of severe illness (exposure) as well as early deaths (outcome) using chi square, ANOVA, and Kruskal-Wallis tests, as appropriate. We identified the potential confounders for the association between exposure and outcome: baseline characteristics associated with exposure (unadjusted *P*<.05) as well as outcome (unadjusted *P*<.20). Age and sex were considered irrespective of their unadjusted *P* values.

We assessed the association between the key exposure variable and outcome using unadjusted and adjusted relative risks (RR and aRR with 95% confidence intervals (CI)). We assessed the confounder-adjusted association using a modified Poisson regression with robust variance estimates (model built using enter method), after ruling out multicollinearity.

We calculated the adjusted incidence proportion (predicted probabilities) for those with and without exposure.

For a power of 80% and alpha error of 5%, the sample size of exposed and unexposed patients in our study was sufficient to detect at least a 3.2% absolute difference in the incidence proportion of early deaths.

### Ethics

We received ethics approval from the Institute Ethics Committee of Employees’ State Insurance Corporation Medical College and PGIMSR, Bengaluru, India (532/L/11/12/Ethics/ESICMC&PGIMSR/Estt.Vol.IV) and the Ethics Advisory Group of the International Union Against Tuberculosis and Lung Disease (The Union), Paris, France (EAG 36/2020). We used verbal consent (rather than written consent) to mimic the implementation in routine program settings and enable the assessment of feasibility, which was approved by the ethics committees.

## RESULTS

### Baseline Characteristics

Of 3,020 people with TB, we excluded 10 due to an incorrect diagnosis. Of 3,010 included in the analysis, 1,529 (50.8%) were screened at diagnosis/notification; of them, 537 (35.1%, 95% CI=32.8, 37.6) had a high risk of severe illness (Table 1).

**TABLE 1.** Characteristics of People With TB Notified by Public Health Facilities of 16 Districts in Karnataka, India, October 15 to November 30, 2020, Stratified by High Risk of Severe Illness Status^a^

**Baseline Characteristics** ^b^		**High Risk of Severe Illness at Baseline** **(Exposure)**			**Unadjusted *P* Value for Association With** ^c^	
	**Total, No. (%) (N=3,010)** ^b^	**Exposure, No. (%)** (n=537)	**No Exposure, No. (%)** (n=992)	**Unknown, No. (%)** (n=1,481)	**Exposure**	**Outcome** ^d^
**Demographic**						
Age, years						
15–24	475 (15.8)	79 (14.7)	155 (15.6)	241(16.3)	.88	<.01
25–34	609 (20.2)	112 (20.9)	192 (19.4)	305 (20.6)		
35–44	617 (20.5)	112 (20.9)	209 (21.1)	296 (20.0)		
45–54	573 (19.0)	93 (17.3)	197 (19.9)	283 (19.1)		
55–64	413 (13.7)	74 (13.8)	132 (13.3)	207 (14.0)		
≥65	323 (10.7)	67 (12.5)	107 (10.8)	149 (10.1)		
Means (SD)	41.8 (15.8)	42.3 (16.6)	42.0 (15.4)	41.6 (15.8)	.17	<.01
Gender						
Men	2,033 (67.5)	364 (67.8)	679 (68.4)	990 (66.8)	.70	<.01
Women	977 (32.5)	173 (32.2)	313 (31.6)	491 (33.2)		
District					<.01	.33
**Clinical**						
Test used for diagnosis						
Rapid molecular	1,623 (53.9)	291 (54.2)	496 (50.0)	836 (56.4)	<.01	.49
Microscopy/culture	564 (18.7)	99 (18.4)	195 (19.7)	270 (18.2)		
Chest radiograph	272 (9.0)	65 (12.1)	103 (10.4)	104 (7.0)		
Others^e^	551 (18.3)	82 (15.3)	198 (20.0)	271 (18.3)		
Bacteriological confirmation (yes)	2,275 (75.6)	418 (77.8)	715 (72.1)	1,142 (77.1)	.01	.92
Site						
Pulmonary	2,185 (72.6)	446 (83.1)	763 (76.9)	976 (65.9)	<.01	<.01
Extra-pulmonary	667 (22.2)	90 (16.8)	228 (23.0)	349 (23.6)		
Missing	158 (5.2)	1 (0.2)	1 (0.1)	156 (10.5)		
Previous treatment (yes)	473 (15.7)	111 (20.7)	137 (13.8)	225 (15.2)	<.01	.38
HIV						
Positive	225 (7.5)	37 (6.9)	72 (7.3)	116 (7.8)	<.01	<.01
Negative	2,526 (83.9)	483 (89.9)	874 (88.1)	1,169 (78.9)		
Unknown	259 (8.6)	17 (3.2)	46 (4.6)	196 (13.2)		
DM						
Yes	505 (16.8)	83 (15.5)	191 (19.3)	231 (15.6)	<.01	<.01
No	2,084 (69.2)	413 (76.9)	724 (73.0)	947 (63.9)		
Unknown	421 (14.0)	41 (7.6)	77 (7.8)	303 (20.5)		
**Health system related**						
Bank details available (yes)	2,352 (78.1)	443 (82.5)	837 (84.4)	1,072 (72.4)	<.01	<.01
Peripheral health institute–notification facility						
District/teaching	1,937 (66.6)	274 (54.9)	572 (59.3)	1,091 (75.4)	<.01	.46
Subdistrict level	767 (26.4)	192 (38.5)	325 (33.7)	250 (17.3)		
Primary level	206 (7.1)	33 (6.6)	67 (7.0)	106 (7.3)		
Time interval between diagnosis and starting treatment						
Within 1 day	1,758 (58.4)	313 (58.3)	582 (58.7)	863 (58.3)	.02	<.01
2–6 days	997 (33.1)	191 (35.6)	337 (34.0)	469 (31.7)		
7 days and longer	255 (8.5)	33 (6.1)	73 (7.3)	149 (10.1)		
Median (IQR)	1 (0.3)	1 (0.3)	1 (0.3)	1 (0.3)	.67	<.01

Abbreviations: DM, diabetes mellitus; IQR, interquartile range; SD, standard deviation.

a Age ≥15 years, all forms, without known drug-resistant disease at diagnosis.

b Source is the routinely collected baseline data in NIKSHAY updated as on December 15, 2020.

c Chi square test for categorical variable, ANOVA for age (continuous data), Krushkal Wallis for time interval (continuous data).

d Early death (within 2 months).

e Includes instances where test of diagnosis was missing.

### Incidence of Early Deaths

**FIGURE 1:**
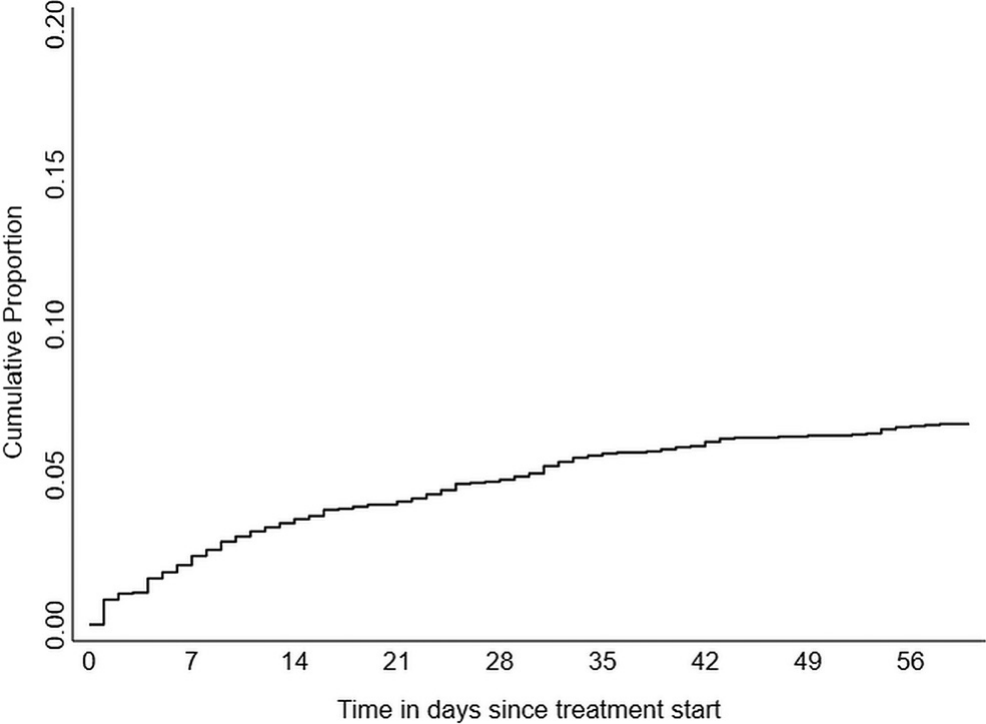
Kaplan-Meier Failure Curve Depicting the Cumulative Proportion of Early Deaths (Within 2 Months) Over Time Among People With TB^a^ Notified by Public Health Facilities in 16 Districts in Karnataka, India From October 15 to November 30, 2020, N=3,010 ^a^ Age ≥15 years, all forms, without known drug-resistant disease at diagnosis.

Among 3,010 people, a total of 195 (6.5%, 95% CI=5.7, 7.4) early deaths were reported with an incidence rate of 3.5 per 100 person-months (95% CI=3.1, 4.1). We have depicted the distribution of these 195 early deaths in Table 2 and Figure 1. The median duration from treatment start to early death was 13 days (interquartile range: 5, 30). Fifty-nine (30.2%) early deaths happened within a week, 100 (51.3%) within 2 weeks, and 140 (71.8%) within 4 weeks (Table 2). This was reflected in the high incidence rate in the initial days of treatment: 24.9 per 100 person-months for the “0–1 day” cohort and 10 per 100 person-months for the “0–6 day” cohort (data not shown).

**TABLE 2.** Distribution of Early Deaths (Within 2 Months) Among People With TB^a^ Notified by Public Health Facilities of 16 Districts in Karnataka, India, October 15 to November 30, 2020

**Time Period **	**No. (%) (N=195)**	**Cumulative % **
Within 1 day	25 (12.8)	12.8
2–6 days	34 (17.4)	30.3
7–13 days	41 (21.0)	51.3
14–27 days	40 (20.5)	71.8
28 days and longer	55 (28.2)	100

a Age ≥15 years, all forms, without known drug-resistant disease at diagnosis.

Of 195 early deaths, 45 (23.1%) were among women. The mean age of TB patients who died was 48.2 years (standard deviation: 16.8); it was significantly lower among women when compared to men (mean 42.4 versus 50.0 years, *P*=.008).

At the end of 2 months, 2,687 (89.3%) were in treatment, 53 (1.8%) were reported lost to follow-up (24 pre-treatment and 29 during treatment), and 75 (2.4%) were transferred to drug-resistant TB care.

### Identification of Potential Confounders

We have depicted the association between baseline characteristics and early deaths and between high risk of severe illness and early deaths in Table 1.

Early deaths were associated with age, gender, site of TB, HIV status, diabetes mellitus (DM) status, availability of bank details, and delay in treatment initiation (Table 1). Patients without bank accounts are mostly from marginalized populations (for example, the migrant poor) and are at a higher risk of death due to the association of poverty with malnutrition. The mean age was significantly higher among patients who died early when compared to those who did not (48.2 versus 41.4 years). Early deaths were significantly higher in men when compared to women (7.4% versus 4.6%) and among patients whose TB site was not recorded (27.8%) when compared to those with pulmonary (5.2%) and extrapulmonary TB (5.7%). Early deaths were also significantly higher among patients with HIV (13.3%) and unknown status (12.7%) when compared to patients without HIV (5.2%) and among patients with unknown DM status (14.0%) when compared to those with DM (6.5%) and those without DM (4.9%).

Early deaths were seen in 7% of patients; deaths were significantly higher among people with a high risk of severe illness.

We identified age, sex, site of TB, HIV, DM, availability of bank details, and treatment initiation delay (from diagnosis) as potential confounders for the association between high risk of severe illness and early death.

### Association Between High Risk of Severe Illness and Early Deaths

**FIGURE 2:**
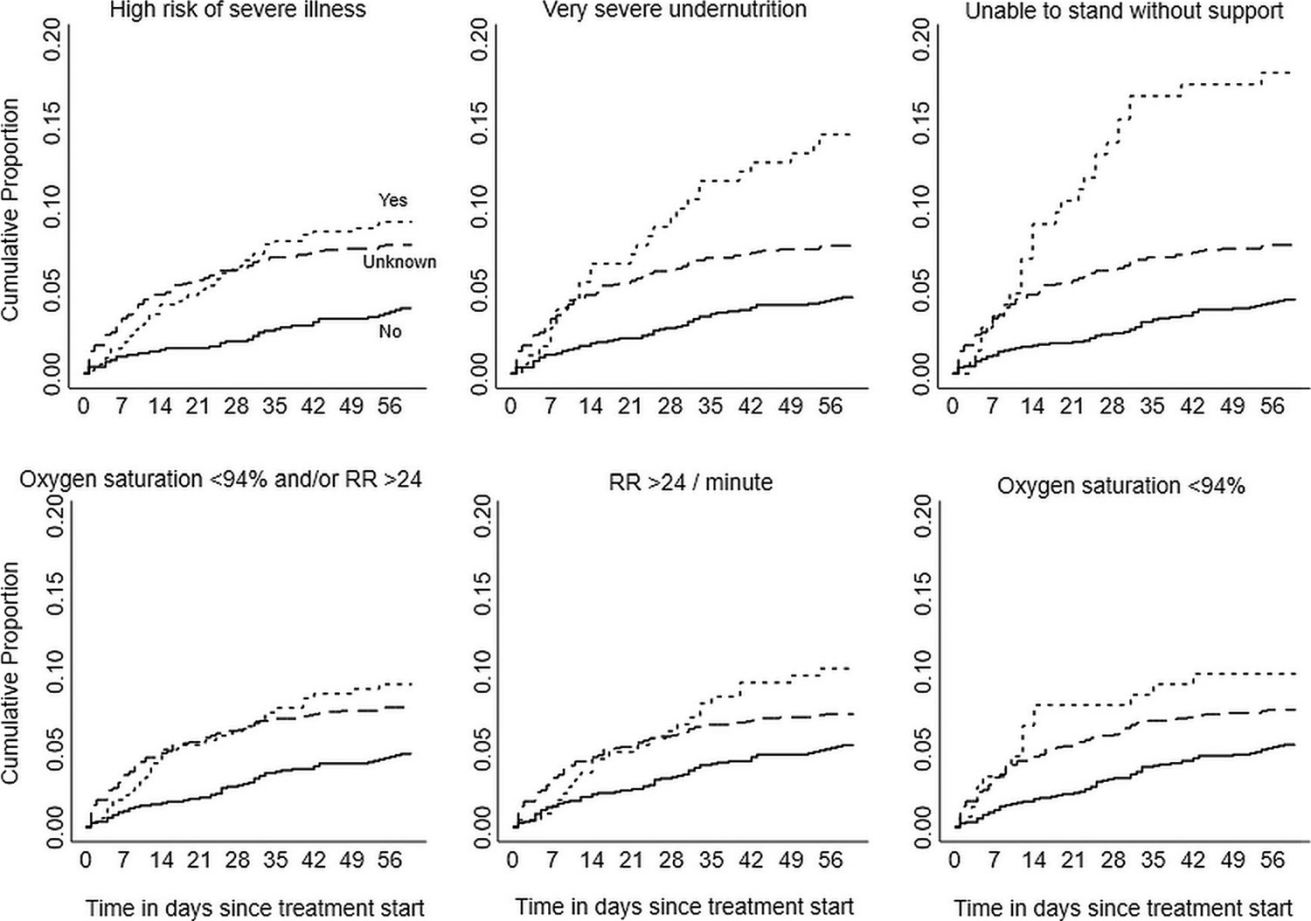
Kaplan-Meier Failure Curves Depicting the Cumulative Proportion of Early Deaths (Within 2 Months) Over Time, Stratified by High Risk of Severe Illness Among People With TB^a^ Notified by Public Health Facilities in 16 Districts in Karnataka, India From October 15 to November 30, 2020, N=3,010 Abbreviation: RR, respiratory rate. Note: Very severe undernutrition is body mass index <14 kg/m^2^ or 14–15.9 with bilateral leg swelling. Log rank P value <0.05 for all. ^a^ Age ≥15 years, all forms, without known drug-resistant disease at diagnosis.

We have depicted the incidence proportion and cumulative proportion of early deaths over time stratified by a high risk of severe illness status in Table 3 and Figure 2. The incidence proportion of early deaths was 8.9% (95% CI=7.5, 12.8) among those with a high risk of severe illness and 3.8% (95% CI=2.8, 5.2) among those without. It was as high as 17.6% among those unable to stand without support and 14.1% among those with very severe undernutrition (Table 3). It was between 4% and 5% for other BMI categories: 14–15.9 without leg swelling, 16–18.4, and ≥18.5 kg/m2 (data not shown).

**TABLE 3.** Association (Unadjusted) Between Death and High Risk of Severe Illness and Its Individual Components Among People With TB^a^ Notified by Public Health Facilities of 16 Districts in Karnataka, India, October 15 to November 30, 2020

		**Incidence**			
	**Total, N=3,010**	**Deaths, No. (%)**	**Rate per 100 pm** ^b^	**RR** ^c^	**95%CI**
		195 (6.5)	3.5	-	
High risk of severe illness					
Yes	537	48 (8.9)	4.8	2.33	1.54, 3.52
Unknown	1481	109 (7.4)	4.2	1.92	1.34, 2.75
No	992	38 (3.8)	2.0	Ref	
BMI <14kg/m^2^ or 14–15.9 with leg swelling					
Yes	184	26 (14.1)	7.9	3.15	2.04, 4.86
Unknown	1488	109 (7.3)	4.2	1.63	1.20, 2.22
No	1338	60 (4.5)	2.4	Ref	
Unable to stand without support					
Yes	148	26 (17.6)	10.3	4.04	2.64, 6.20
Unknown	1481	109 (7.4)	4.2	1.69	1.25, 2.30
No	1381	60 (4.3)	2.3	Ref	
Respiratory rate >24/min and/or oxygen saturation <94%					
Yes	345	31 (9.0)	4.9	1.95	1.27, 2.98
Unknown	1494	110 (7.4)	4.2	1.60	1.16, 2.19
No	1171	54 (4.6)	2.4	Ref	
Respiratory rate >24/min					
Yes	232	23 (9.9)	5.5	1.92	1.21, 3.03
Unknown	1580	110 (7.0)	3.9	1.35	0.99, 1.82
No	1198	62 (5.2)	2.7	Ref	
Oxygen saturation <94%					
Yes	155	15 (9.7)	5.4	1.86	1.09, 3.17
Unknown	1569	113 (7.2)	4.1	1.38	1.03, 1.85
No	1286	67 (5.2)	2.7	Ref	

Abbreviations: BMI, body mass index; CI, confidence interval; PM, person-months; RR, relative risk.

a Age ≥15 years, all forms, without known drug-resistant disease at diagnosis.

b Incidence of deaths per 100 person-months of follow up.

c RR calculated as a ratio of incidence proportions.

After adjusting for potential confounders, people with a high risk of severe illness had a significantly higher risk of early death when compared to those without (aRR: 2.36; 95% CI=1.57, 3.55). Adjusted incidence proportion of early deaths was 11.2% (95% CI=8.1, 14.2) among those with a high risk of severe illness and 4.7% (95% CI=3.2, 6.2) among those without (data not shown).

## DISCUSSION

Screening for severe illness at diagnosis/notification and the association of severe illness with early TB death in programmatic settings is not well studied in India and other high-burden countries. To the best of our knowledge, our study is the first of its kind. Except for a single 2005–2009 study from Andhra Pradesh (which reported that 50% of early deaths occurred within the first 4 weeks of treatment, compared to 75% in our study), limited studies from India have documented the timing of TB deaths.^28^

There were 3 relevant findings. First, the incidence proportion of early deaths (6.5%) was similar to the incidence proportion of total deaths reported during treatment (7%) for the 2018 cohort in Karnataka.^27^ This and the increased share of early deaths in the first month when compared to Andhra Pradesh (2005–09)^28^ indicates an increase in fatality rate during the COVID-19 pandemic when compared to the pre-pandemic period.

Second, half of the early deaths happened within 2 weeks; early deaths were significantly associated with a high risk of severe illness. However, the incidence in the first week in our study (10 per 100 person-months) was lower than the 37.6 per 100 person-months reported by Nigeria (2010–14).^8^ Nonetheless, a significant proportion of early deaths within the first 2 weeks reinforces the need for indicators that are easy to use and interpret and can be applied at diagnosis in primary care settings. Our experience here in Karnataka as well as in Gujarat (a state in west India) shows that the indicators used in our study can be measured within a few days of diagnosis/notification and are therefore potentially effective in preventing early deaths.^23^^,^^29^ This also underlines the need for increased vigilance during the first few weeks and to counsel patients and their caretakers to report to the health program staff any warning signs, like inability to stand without support or symptoms of breathlessness.

The indicators used in this study can be measured within a few days of diagnosis/notification and are therefore potentially effective in preventing early TB deaths.

A study from South India (2018–2019) recommended differentiated TB care (in the form of intensive treatment support) for people with any 1 of the following risk factors: age ≥60 years, living alone, HIV, DM, previous treatment, drug-resistant TB, regular alcohol consumption, and undernutrition (weight less than 43 kg for men and 38 kg for women). In the project setting, this assessment of risk was completed at around 2 months, possibly due to delays in laboratory-based HIV, DM, and drug susceptibility testing.^30^ The risk assessment indicators used in this study were comprehensive but may be challenging in current programmatic settings. The resultant delay in severity assessment and identification is likely to miss early TB deaths.

Third, this study was an effort to assess the feasibility of screening and quantify the association of high risk for severe illness with early death. We used indicators related to very severe undernutrition, respiratory insufficiency, and performance status. Due to a lack of policy guidance at the time of our study, screen-positive patients were not systematically referred and tracked for appropriate care. Screening alone does not produce better outcomes. Screening for severe illness at diagnosis/notification can prevent early deaths if we implement this as a routine activity and link screen-positive patients to appropriate care.

We recommend that high risk of severe illness (yes/no) be recorded as a baseline characteristic in NIKSHAY. This will require the availability of tools to measure height at all peripheral health facilities, which is still a challenge in India. Once heights are measured accurately, the program has tools to enable BMI estimation.^31^ Those with a high risk of severe illness may be prioritized for referral to higher facilities for comprehensive clinical evaluation and inpatient care. This will require systems of referral, availability of adequate beds for inpatient care, and staff trained in the management of severely ill patients with TB and its comorbidities. Inpatient care should include the care of severe disease, complications, and comorbidities such as HIV and DM. As the inability to stand without support and very severe undernutrition had relative higher incidences of early deaths, inpatient clinical care should also focus on therapeutic nutrition, for which guidance is available.^15^

### Future Research

We have reported only early TB deaths; we will report on the mortality experience at the end of treatment and recurrence post-treatment in a subsequent analysis. We used indicators that are easily measurable and simple to interpret by paramedical staff at the time of diagnosis/notification. While adding additional indicators may improve the sensitivity of the tool, it may make it impractical for paramedical program staff to apply in the field and may contribute to delay. Future studies should document the accuracy of this screening tool as applied by health professionals, paramedics, and community health workers so that the NTP has an idea of how many severely ill people with TB are missed or misclassified when different kinds of personnel use the tool. In addition, further operational research is required to assess the feasibility and impact of systematic referral and inpatient care.

Future studies should document the accuracy of this screening tool as applied by health professionals, paramedics, and community health workers.

### Limitations

There were a few major limitations. First, of the 3,010 TB cases registered during the period, we were not able to screen 1,481 (49%) TB patients for high risk of severe illness. This was due to the ongoing COVID-19 pandemic that had distressed the health system. We decided to retain the “unknown” status (those not screened at baseline) as 1 of the 3 exposure categories. Those with “unknown” exposure status (7.4%) experienced early deaths similar to those who were classified as being at high risk of severe illness (8.9%). The program was missing a large number of individuals with a high risk of severe illness among those not screened. This makes a strong case for improving the coverage of screening for severe illness. Second, due to the pandemic, we were not able to conduct in-person trainings and perform a reliability assessment of the measurements. However, as reported previously, the observed data quality was acceptable for a program setting.^23^

Third, people with a high risk of severe illness were not systematically referred to higher facilities for evaluation due to a lack of guidance/policy from the state at the time of our study. We were also not able to ascertain patient management regarding HIV infection, severe undernutrition, and DM. However, there are reasons to believe that some local action might have been taken to provide care to these patients. Hence, our estimate of the association between high risk of severe illness and early death is an underestimate.

Fourth, the COVID-19 response-related lockdown resulted in 39% TB under-detection in the 16 study districts during the study period; half the detected TB patients were not assessed for severe illness.^32^ It is possible that only those who were severely ill sought care, which can lead to over-estimation of the burden, as well as the higher occurrence of early deaths in this cohort. On the other hand, the limited number of indicators used to identify severe illness might also have led to an underestimation of the real burden.

Finally, at the time of our study, the program did not capture the COVID-19 status of TB patients; therefore, we do not have data on COVID-19 co-infection.

## CONCLUSION

In this operational research from Karnataka, India, routine TB program staff screened people with TB for severe illness using indicators of very severe undernutrition, abnormal vital signs, and poor performance status. These indicators are simple to use and easy to interpret without the need for diagnostic infrastructure and clinical capacity. We documented the association of high risk of severe illness at baseline with early deaths. A high burden of severe illness and strong association with early deaths indicates that systematic screening, referral, and inpatient care of severely ill patients at diagnosis/notification has the potential to detect a large number of people who are likely to experience early deaths. Screening for severe illness in people with TB at the time of diagnosis/notification is low-hanging fruit in achieving the 2030 TB deaths target of Sustainable Development Goals and WHO by 2025 in India.^2^^,^^26^

## Supplementary Material

GHSP-D-21-00736-supplement-2.pdf

GHSP-D-21-00736-supplement-3.xlsx

GHSP-D-21-00736-supplement-1.pdf

## Notes

**Peer Reviewed**

**First published online:** August 4, 2022.
