# Supplementary material for: Screening for Severe Illness at Diagnosis Has the Potential to Prevent Early TB Deaths: Programmatic Experience From Karnataka, India
Source: Glob Health Sci Pract. 2022 Aug 30;10(4):e2100736. doi: 10.9745/GHSP-D-21-00736 (PMC9426979; doi:10.9745/GHSP-D-21-00736)
Supplement: GHSP-D-21-00736-supplement-1.pdf [file GHSP-D-21-00736-supplement-1.pdf]

Supplement to: Shewade HD, Nagaraja SB, Vanitha B, et al. Screening for severe illness at diagnosis has the potential to prevent early TB deaths: programmatic experience from Karnataka, India. Glob Health Sci Pract. 2022;10(4):e2100736. https://doi.org/10.9745/GHSP-D-21-00736

STANDARD OPERATING PROCEDURE –

Screening notified TB patients at public PHI for 'high risk of severe illness'

Purpose: The purpose of this SOP is to outline the procedure to be followed for screening for 'high risk of severe illness'

Responsibility: At public PHI, the lab technician should file the paper-based data collection tool. He may be supported by the TB-HV. Final responsibility of this lies with the lab technician. Please take support of Medical Officer or STS in correct filling of the paper-based data collection tool. The hard copy should be filed in public PHI by lab technician.

WEIGHT:

- Weight to be recorded after removing heavy warm clothing, belt and shoes
- Use the available weighing machine.
- The participant is be made to stand still and upright with weight evenly distributed between the two feet.
- Remember to ask the participant to not look down to check his or her own reading so as to measure correct reading.
- Take 2 readings if needed and enter the average in the paper based data collection tool (weight in kg, max one decimal point).

HEIGHT:

- Height to be measured using a portable stadiometer. It has to be kept on an even surface.
- The participant to be instructed for the following:
- Remove foot wear.
- Remove buns and headgears that interfere with measurement.
- Ensure contact of 4 points: head, shoulder blade, buttocks and heels.
- Weight to be borne evenly on both feet, heels almost together with feet pointing outwards at 60 degree angle.
- Rest the Stadiometer headpiece on the head to just compress the hair.
- Ask the person to take a deep breath and hold it: this straightens the spine and makes the measurement reproducible.
- If the participant is taller than the examiner, use a stool.
- Record the readings in the paper-based data collection tool (height in cm, no decimal point)

BODY MASS INDEX (BMI):

- Convert height in cm into height in metre (max two decimal point). For ex 170 cm is 1.70 m, 152 cm is 1.52 m. Calculate the square of height in meters. For ex for 1.70, the square is 1.70*1.70= 1.89. For 1.52 m, the square is 1.52*1.52 = 2.31. Then weight in kg divided by height in meter square will be the BMI
- Some examples have been given below
- Enter the BMI in paper based data collection tool (max one decimal point)

**Weight (kg)**	**Height (cm)**	**Height (m)**	**Height in metre square (m^2)**	**BMI (kg / m^2)**
60	170	1.70	1.70*1.70 = 2.89	60/2.89 = 20.8
60	152	1.52	1.52*1.52 = 2.31	60/2.31 = 26.0
50	170	1.70	1.70*1.70 = 2.89	50/2.89 = 17.3
50	152	1.52	1.52*1.52 = 2.31	50/2.31 = 21.6
40	170	1.70	1.70*1.70 = 2.89	40/2.89 = 13.8
40	152	1.52	1.52*1.52 = 2.31	40/2.31 = 17.3

SWELLING OF LIMBS

- Expose the patient’s legs and examine them with the patient standing/lying supine.
- Press gently with your fingertip over the bony prominence 2cm above the ankle for a few seconds and then see if your finger has left a pit.
- Remember to avoid areas with wounds.

RESPIRATORY RATE:

- Quietly observe the participant’s breathing pattern without drawing their attention to it, as this may cause it to change.
- Sleeping position may be considered for easy count
- Count the number of times the participant inhales (inhalation and exhalation is 1 full count) for a full 1 minute.
- Record the number of breaths per minute in the paper based data collection tool.

PULSE OXIMETRY

A clip-like device called a probe is placed on the index finger. The probe uses light to measure how much oxygen is there in the blood.

- Normal should be 94% and above
- If less than 94% please check on the fingers of your own hand and the other hand of the patient
- If less than 94% the patient must be assessed by the medical officer

If less than 90, consider it as a medical emergency and facilitate admission of the patient in the nearest government hospital. After recording enter the reading in the paper based tool.
